# Trem-2 Promotes Emergence of Restorative Macrophages and Endothelial Cells During Recovery From Hepatic Tissue Damage

**DOI:** 10.3389/fimmu.2020.616044

**Published:** 2021-02-08

**Authors:** Inês Coelho, Nádia Duarte, André Barros, Maria Paula Macedo, Carlos Penha-Gonçalves

**Affiliations:** ^1^ Instituto Gulbenkian de Ciência, Oeiras, Portugal; ^2^ CEDOC, NOVA Medical School/Faculdade de Ciências Médicas, Universidade Nova de Lisboa, Lisboa, Portugal; ^3^ APDP Diabetes Portugal, Education and Research Center (APDP-ERC), Lisbon, Portugal; ^4^ Department of Medical Sciences, Institute of Biomedicine - iBiMED, University of Aveiro, Aveiro, Portugal

**Keywords:** triggering receptor expressed on myeloid cells 2, macrophages, tissue repair and organ regeneration, inflammation, carbon tetrachloride 4, acetaminophen (paracetamol), endothelial cells

## Abstract

Macrophages are pivotal in mounting liver inflammatory and tissue repair responses upon hepatic injury, showing remarkable functional plasticity. The molecular mechanisms determining macrophage transition from inflammatory to restorative phenotypes in the damaged liver remain unclear. Using mouse models of acute (APAP) and chronic (CCl4) drug-induced hepatotoxic injury we show that the immune receptor Trem-2 controls phenotypic shifts of liver macrophages and impacts endothelial cell differentiation during tissue recovery. Trem-2 gene ablation led to a delayed re-population of Kupffer cells correlating with deterred resolution of hepatic damage following acute and chronic injury. During tissue recovery, we found that macrophages transitioning to Kupffer cells expressed high levels of Trem-2. Acquisition of the transition phenotype was associated with a unique transcriptomic profile denoting strong responsiveness to oxidative stress and downmodulation of the pro-inflammatory phenotype, which was not observed in absence of Trem-2. During tissue recovery, lack of Trem-2 favored accumulation of a liver-damage associated endothelial cell population (LDECs), whose transcriptional program was compatible with endothelial de-differentiation. Accordingly, LDECs precursor potential is supported by the downregulation of surface endothelial cell markers and by striking *in vitro* morphological changes towards typical endothelial cells. In conclusion, we found that the dynamics of liver macrophages in response to liver injury are critically controlled by Trem-2 and this regulation is interlinked with the de-differentiation of endothelial cells and heightened liver pathology. We propose that Trem-2 promotes the transition from pro-inflammatory to tissue repair phase by driving the acquisition of restorative properties in phagocytic macrophages.

## Introduction

Hepatotoxic insults elicit a multilayered response involving damaged tissue clearance, scar formation and tissue regeneration. Macrophages play decisive roles in inflammatory and tissue repair responses during acute and chronic liver injury (1–3) as well as in liver damage due to metabolic disorders such as NAFLD (4, 5), type 2 diabetes and obesity (6).

In damaged hepatic tissue, macrophages with different surface phenotypes and activation status show sharp population dynamics (1, 3) suggesting that distinct macrophage populations perform specific activities that determine the course of response to tissue damage. Macrophage involvement in response to severe damage is often initiated by influx of hematopoietic-derived monocytes that home the liver as Ly6c^+^ cells and dominate the liver macrophage populations at this stage (2, 3, 7). These Ly6c^+^ cells present a high-inflammatory phenotype including the expression of TNF-α, IL-1β and TGF-β signals that amplify tissue pathology and also, in chronic tissue injury promote transdifferentiation of stellate cells into collagen-producing myofibroblasts, a hallmark of liver fibrosis (1).

Conversely, tissue repair and fibrosis resolution are associated with the emergence of macrophages that phenotypically resemble liver resident macrophage cells (1, 3). These pro-resolution macrophages show phagocytic ability and express high levels of metalloproteinases and anti-inflammatory mediators (e.g. MMP12 and Arg1) that trigger myofibroblasts apoptosis and actively participate in extracellular matrix degradation (1). Still, however, it is unclear what regulates the dynamics of different liver macrophage populations during response to damage. Macrophages show remarkable phenotypic and functional plasticity and are equipped to undergo functional transitions, depending on contextual cues (8, 9). Interestingly, it has been shown that pro-inflammatory macrophages can acquire anti-inflammatory and pro-repair phenotypes (1, 3, 10) but the triggers for this phenotype switch in liver macrophages remain largely unknown (1, 9).

Triggering receptor expressed on myeloid cells-2 (Trem-2) is a transmembrane immune receptor typically expressed in the monocyte/macrophage lineage (11). Upon ligand binding Trem-2 signals through the adaptor DAP12, thereby modulating activation of macrophage effector functions (12). Trem-2 has been intensively studied in the context of neurodegenerative diseases, revealing its concurrent role in the engulfment of αβ-amyloid plaques during Alzheimer’s disease (13) and in phagocytosis of apoptotic neurons (14). In addition, Trem-2 signaling was shown to limit tissue destruction and to facilitate both repair and cellular debris clearance in a model of Experimental Autoimmune Encephalomyelitis (EAE) (15). Trem-2 ligands leading to macrophage activation *in situ* have not been identified, however various studies have reported on a binding to phospholipids such as phosphatidylserine (14, 16) and a range of acidic and zwitterionic lipids (13), which may accumulate upon cell damage.

Furthermore, Trem-2 has been shown to modulate microglia survival through Wnt/β-catenin signaling (17, 18) and also to promote inhibitory signals that restrain pro-inflammatory macrophage activation (19, 20). Recent studies have also illustrated that Trem-2 is required for the activation of a specific transcriptional gene program which controls phagocytosis and lipid metabolism of microglial cells in Alzheimer’s disease (21) and of lipid associated macrophages (LAM) in metabolic disorders (22).

The impacts of Trem-2 in liver macrophages have been less explored. Previous findings from our laboratory uncovered that Trem-2 is expressed on Kupffer cells (KCs) determining their activation profile upon contact with malaria parasite (23). Recently published work (24) revealed that Trem-2 is involved in liver damage and proposed that Trem-2 expression in non-parenchymal cells acts as a brake of the inflammatory response during hepatotoxic injury. Although this established a link between Trem-2 and liver inflammation, specific effects on macrophages, the critical players in these processes, remain unsettled.

Here we uncovered that upon experimental induction of hepatic injury Trem-2 controls dynamics of liver macrophage populations favoring replenishment of Kupffer cells and consequently promoting tissue damage resolution and regeneration of the hepatic tissue, including the endothelial cell lineage.

## Materials and Methods

### Mice and Experimental Models

All procedures involving laboratory mice were in accordance with national (Portaria 1005/92) and European regulations (European Directive 86/609/CEE) on animal experimentation and were approved by the Instituto Gulbenkian de Ciência Ethics Committee and the Direcção-Geral de Veterinária (the Official National Entity for regulation of laboratory animals usage). Trem2-deficient mice in a C57BL/6 background (19) were kindly provided by Marco Colonna, Washington University School of Medicine, St. Louis, MO. C57BL/6 mice, Trem-2 KO and B6.Actin-GFP mice were bred and housed under a 12-hr light/dark cycle in specific pathogen free housing facilities at the Instituto Gulbenkian de Ciência.

To induce acute liver injury, C57BL/6 and Trem-2 KO male mice with 10 weeks of age were fasted for 15 hours prior to intra-peritoneal injection with 300mg/Kg of acetaminophen (N-acetyl-p-aminophenol (APAP)) (3) (Sigma, St. Louis, MO, USA) in PBS or PBS only. Liver and blood were collected at day 1 (D1) and 3 (D3) after injection.

In the model of chronic liver fibrosis and fibrosis regression, C57BL/6 and Trem-2 KO males with 7–8 weeks of age received PBS or 20%v/v carbon tetrachloride (CCl4, Sigma, St. Louis, MO, USA) in olive oil, administered at 0,4mL/Kg, twice a week during 4 weeks by intra-peritoneal injections (1). Liver and blood were collected at day 1 (fibrosis) or day 3 post-injection (fibrosis regression).

For the *in vivo* phagocytosis experiments, mice were given a retro-orbital injection of 50x10^6^ beads/200uL fluorescent beads (Fluoresbrite YG microspheres 2μm, Polysciences) 3 days after APAP treatment and 1 h prior liver collection.

### Non-Parenchymal Cells Isolation, Flow Cytometry and Cell Sorting

Non-parenchymal cells (NPCs) were isolated from liver lobes by perfusion with Collagenase H (Sigma, St. Louis, MO, USA) followed by density centrifugation as previously described (25, 26). Non-parenchymal cells (NPCs) were immuno-labeled with fluorochrome-conjugated antibodies (eBiosciences and BioLegend) followed by flow cytometry analysis (LSR Fortessa X20™, BD) or cell sorting (FACSAria, BD). Antibodies are listed in ***Supplementary Methods***.

### Histology

Histological analyses were performed in the Histopathology Unit of Instituto Gulbenkian de Ciência. Livers were fixed in 10% formalin and embedded in paraffin. Non-consecutive 3µm sections were stained with hematoxylin-eosin or Mason’s Trichrome and examined under light microscope (Leica DM LB2, Leica Microsystems, Wetzlar, Germany). Necrosis and fibrosis were blindly assessed by a trained pathologist. Necrosis scoring: 0, no necrosis; 1, single cell centrilobular necrosis to centrilobular necrosis without central to central bridging necrosis; 2, centrilobular necrosis with central to central bridging; 3, centrilobular necrosis with central to central bridging and focal coalescent foci of necrosis; 4, centrilobular necrosis with central to central bridging with large coalescent foci of necrosis. Fibrosis was assessed using Ishak system adaptation: 0, no fibrosis; 1, fibrous expansion in some centrilobular areas with or without fibrous septa; 2, fibrous expansion of most centrilobular areas with or without fibrous septa; 3, fibrous expansion of most centrilobular areas with occasional central to central bridging; 4, fibrous expansion in most centrilobular areas with marked central to central bridging; 5, marked bridging (C-C) with occasional nodules; 6: cirrhosis. Images were acquired on a Leica DM LB2 and a commercial Leica High Content Screening microscope.

For immunofluorescence, PFA fixed cells were stained overnight at 4°C with rat anti-mouse F4/80 diluted 1:50 and rabbit anti-mouse caspase-3, diluted 1:500. On the following day sections were washed and incubated with respective secondary antibodies for 1h at room temperature. Images were acquired using a 5x5 tile-scan protocol on a Nikon Ti microscope using a 20x 0.75 NA objective, coupled with an Andor Zyla 4.2 sCMOS camera (Andor, Oxford Instruments) and controlled through Nikon NIS Elements (Nikon). DAPI, F4/80 and caspase-3 were acquired using a DAPI, Cy5 and TRITC filtersets, respectively. Detailed methods for analysis can be found in ***Supplementary Methods***.

### AST/ALT

Serum levels of Aspartate aminotransferase (AST) and Alanine aminotransferase (ALT) were determined by a colorimetric enzymatic assay using the GOT-GPT kit (Spinreact S.A., Spain) according to manufactures’ instructions.

### RNA Isolation and Gene Expression Analysis

NPCs were collected in lysis buffer (RNeasy MiniKit- Qiagen) and total RNA was obtained using RNeasy MiniKit (Qiagen) and converted to cDNA (Transcriptor First Strand cDNA Synthesis Kit, Roche). Cells-to-ct kit (Applied Biosystems) was used to amplify cDNA from sorted cells. Taqman gene expression assays (TNFa Mm00443258_m1, trem2 Mm00451744_m1, Applied Biosystems) and endogenous control GAPDH were used in multiplex Real-Time PCR reactions (ABI QuantStudio-384, ThermoFischer). Results represent relative quantification calculated using the 2-ΔΔCT method and normalized to GAPDH.

## RNA Sequencing

### Cell Sorting and RNA Sequencing Analysis

Macrophage populations and CD45^neg^ SSC^hi^ population were sorted directly into Qiagen RLT lysis buffer. Each sample represents a pooling of 4 mice. Biological replicates were used for each population except for KCs control. Sequencing was performed at the Genomics Unit, Instituto Gulbenkian de Ciência (IGC, Portugal) following a previously established protocol (27). Briefly, RNA was separated from gDNA using a modified oligo-dT bead-based strategy and DNA libraries were prepared using Pico Nextera protocol. Sequencing was performed using NextSeq500- High Output Kit v2 (75 cycles), single-ended, 20 million reads per sample. Detailed methods for analysis can be found in ***Supplementary Methods***.

### Statistics

To analyze differences across genotypes in treated animals we calculated that a sample size of 5 animals per group would be necessary to have 80% power to detect a mean phenotypic difference higher than 9% at 0.05 significance level. Initial experiments detecting statistical differences, were independently replicated, namely at day 3 post treatments, and their cumulative results are shown. We avoid replicating experiments with initial negative results to prevent unnecessary use of animals. Chi-square test was used to analyze proportions of categorical variables while one-way ANOVA was used to compare means within genotypes and two-way ANOVA to compare means between genotypes. Pearson’s correlation was used to test for correlation between two variables and Man-Whitney test was use to compare mean intensity fluorescence ranks. Statistics were calculated with GraphPad Prism version 6.

In ***Supplementary Methods*** section we provide additional details on ***Materials and Methods***.

## Results

### Trem-2 Ablation Deters Tissue Repair Upon Acute Liver Injury

**Figure 1:**
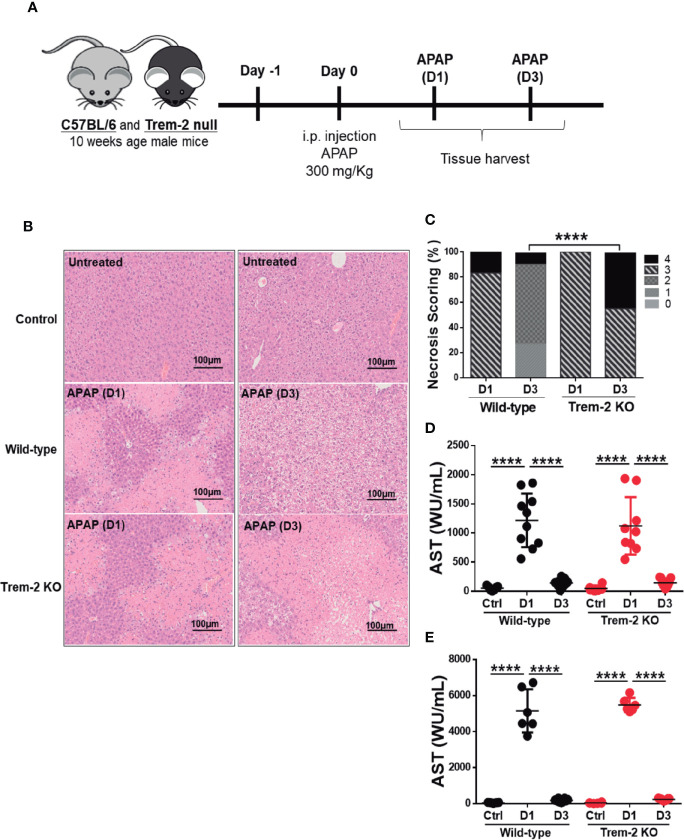
Trem-2 KO mice show impaired recovery from liver damage induced by APAP. Acute liver damage induced by single intra-peritoneal administration of acetaminophen (APAP) was assessed 1 day (APAP-D1) or 3 days (APAP-D3) post-injection of wild-type and Trem-2 KO mice **(A)**. Hepatocyte necrosis was evaluated using hematoxylin-eosin staining **(B)** and scored from 0 to 4 according to location and extension of the necrotic lesions **(C)**. Liver damage was assessed by quantification of hepatic enzymes AST **(D)** and ALT **(E)** in the serum. Mean values and standard deviations are represented. Statistics: Chi-square test in necrosis scoring **(C)**; One-way ANOVA in **(D)**; (n=6-11 mice/group). ****, p<0.0001.

To study the role of Trem-2 in responses to acute liver injury we used a well-established experimental model (3). Mice received a single dose of acetaminophen (APAP) and were analyzed after 1 day (D1) or 3 days (D3), corresponding to the times of hepatic damage and tissue repair responses, respectively (**Figure 1A**). During acute injury (D1) wild-type and Trem-2 KO mice showed regions of massive necrosis in the liver (**Figures 1B, C**). At D3 wild-type had almost completely cleared necrosis while Trem-2 KO mice retained marked liver pathology with wide coalescent necrotic areas (**Figures 1B, C**). Serum levels of hepatic enzymes AST and ALT at day 1 and day 3 and histological analysis at day 1 indicated that the intensity of APAP hepatotoxicity and the extent of tissue damage was not affected by Trem-2 expression (**Figures 1D, E**). Nevertheless, the persistence of larger necrotic areas at day 3 indicate that although wild-type and Trem-2 KO mice were similarly affected by acute liver injury, resolution of liver damage in Trem-2 KO mice was impaired. These data show that although wild-type and Trem-2 KO mice were similarly affected by acute liver injury, resolution of liver damage in Trem-2 KO mice was impaired.

### Trem-2 Impacts on Non-Parenchymal Cells Dynamics During Recovery From Acute Liver Damage

**Figure 2:**
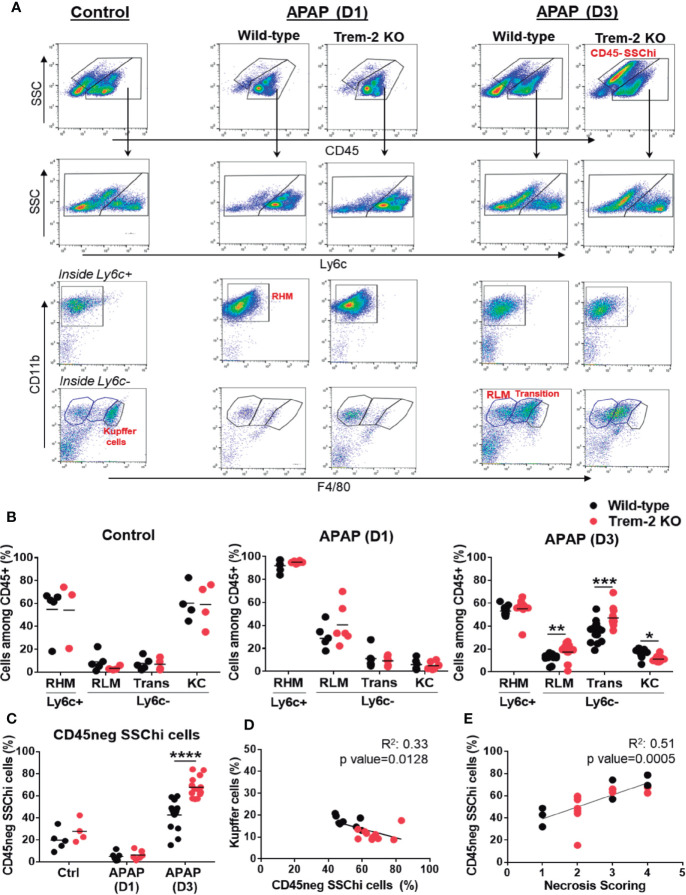
Impaired KC replenishment upon acute liver injury in Trem-2 KO mice and accumulation of non-hematopoietic CD45neg SSChi cells. Flow cytometry analysis of liver non-parenchymal cells (NPCs) from wild-type and Trem-2 KO mice (control versus D1 versus D3) revealed distinct macrophage populations and a non-hematopoietic cell population (CD45neg SSChi). Among macrophages we identified Ly6c+ recruited hepatic macrophages (RHM, CD45+ Ly6c+ F4/80low CD11b+) and Ly6c- macrophages, namely, Kupffer cells (KC, CD45+ Ly6c- F4/80high CD11bint); transition macrophages (Trans, CD45+ Ly6c- F4/80int CD11bhigh); and recruited-like macrophages (RLM, CD45+ Ly6c- F4/80low CD11b+) **(A)**. Cell frequencies of macrophage populations **(B)** and non-hematopoietic CD45neg SSChi population **(C)** in control, APAP-D1 and APAP-D3 wild-type and Trem-2 KO mice. Inverse correlation of KC frequency with CD45neg SSChi population at APAP-D3 **(D)**. Correlation of CD45neg SSChi cells frequency with necrosis scoring at APAP-D3 **(E)**. Symbols represent values from individual mice (n=6-11 mice/group). Group mean values are presented. Statistics: Two-way ANOVA in **(B**, **C)** Pearson’s correlation test in **(D**, **E)** *, p<0.05 **, p<0.01 ***, p<0.001 ****, p<0.0001.

Given the role of Trem-2 in macrophage functional activation (19) we isolated non-parenchymal cells (NPCs) from APAP-treated mice at the time points of liver injury (D1) and tissue repair (D3) and performed a detailed analysis of the macrophage lineage cells populations (**Figure 2A**). Recruited hepatic macrophages (RHM) (CD45^+^ Ly6c^+^ F4/80^int^ CD11b^high^) known to promote tissue inflammation (7, 28) were predominant at D1 but declined during the tissue repair phase (D3). RHM were found in similar proportions in wild-type and Trem-2 KO mice, suggesting that macrophage recruitment to the liver was not affected in absence of Trem-2 (**Figures 2A, B**). As expected, Kupffer cells (KCs) (CD45^+^ Ly6c^-^ F4/80^hi^ CD11b^int^) were highly represented in untreated mice and were severely reduced during injury (D1) in wild-type and Trem-2 KO mice (**Figures 2A, B**). However, we noted that in the tissue repair phase (D3) the recovery of KCs was slower in Trem-2 KO mice (**Figures 2A, B** and **Supplementary Figure 1**). We have recently shown that levels of CD26 enzymatic activity, from unidentified cellular sources, is a serum biomarker that mirrors severe reductions in KCs population (26). Quantification of CD26 activity in the serum showed that at D3 Trem-2 KO mice reach slightly higher levels of CD26 activity (**Supplementary Figure 2A**) corroborating the delayed KCs replenishment in Trem-2 KO mice.

Interestingly, our analysis revealed two related liver Ly6c^-^ macrophage populations with distinctive surface phenotypes: a CD45^+^ Ly6c^-^ F4/80^low^ CD11b^+^ population enriched at D1 and resembling the typical recruited Ly6c^+^ counterpart, herein named as recruited-like macrophages (RLM) (**Figure 2A**) and a CD45^+^ Ly6c^-^ F4/80^+^ CD11b^hi^ population showing a surface phenotype close to KCs and named as transition macrophages that was strikingly enriched during tissue repair (D3) (**Figures 2A, B**). In contrast to KCs these populations accumulated in Trem-2 KO mice at D3 (**Figure 2B**) indicating that in absence of Trem-2 dynamics of macrophage hepatic repopulation was altered during tissue repair response.

Using GFP-labeled monocyte transfers we show that after acute liver injury recruited bone-marrow monocytes give rise to RLM, transition macrophages and KCs (**Supplementary Figure 3**). Together, these results suggest that in response to acute liver damage Trem-2 is a determinant of macrophage population dynamics promoting the replenishment of the KCs niche from recruited monocytes.

In addition, flow cytometry analysis uncovered a previously unnoticed non-hematopoietic CD45^neg^ SSC^hi^ population that emerges at D3 and accumulates in Trem-2 KO mice (**Figures 2A, C**) paralleling the accumulation of transition macrophages. Strikingly, we noted that accumulation of non-hematopoietic CD45^neg^ SSC^hi^ cells inversely correlate with the KCs proportion in the livers of wild-type and Trem-2 KO mice (**Figure 2D**) and directly correlated with the persistence of liver necrosis (**Figure 2E**). These findings suggest that in absence of Trem-2, imbalanced replenishment of liver macrophages associated with accumulation of transition macrophages results in overrepresentation of a non-parenchymal cell population, which correlates with impaired resolution of liver necrosis.

### Trem-2 Ablation Delays Tissue Repair and Alters Non-Parenchymal Cell Dynamics in Chronic Liver Damage

**Figure 3:**
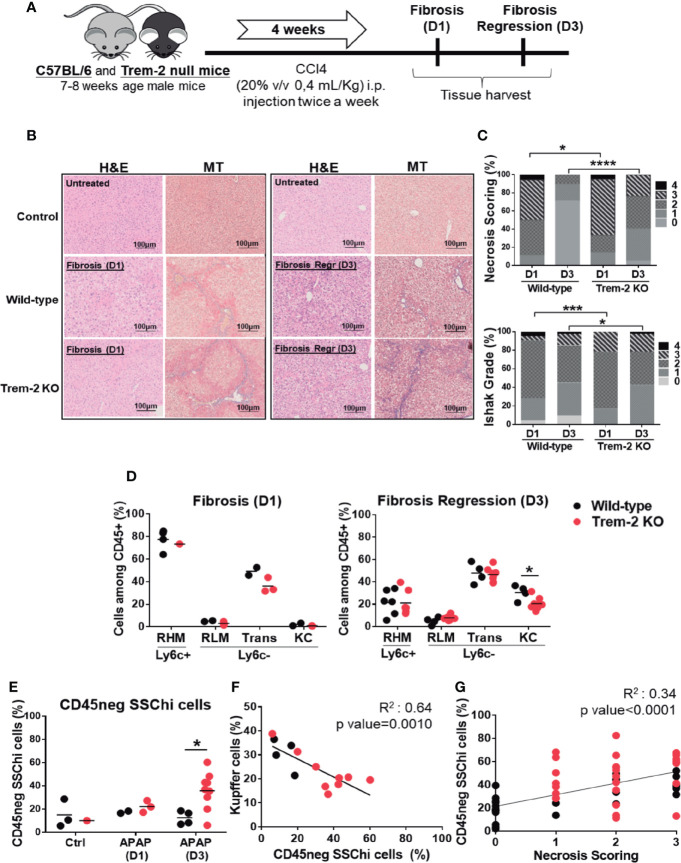
Responses to chronic liver injury are impaired in Trem-2 KO mice. Chronic liver injury was induced by administration of CCl4 during 4 weeks, twice a week. Mice were analyzed one or three days after the last injection, corresponding to fibrosis-D1 and fibrosis regression-D3 time points in wild-type mice **(A)**. Liver necrosis was evaluated using hematoxylin-eosin (H&E) staining **(B)** and scored from 0 to 4 according to location and extension of the necrotic lesions **(C)**. Masson’s trichrome (MT) staining was used to identify and quantify collagen fibers (blue) **(B)** using an adapted Ishak grading **(D)**. Applying the flow cytometry criteria depicted in **Figure 2** we quantified cell frequencies of distinct macrophage populations: recruited hepatic macrophages (RHM); Kupffer cells (KC); transition macrophages (Trans); recruited-like macrophages (RLM) **(E)** and of non-hematopoietic CD45neg SSChi population **(F)** in wild-type and Trem-2 KO mice for control, fibrosis-D1 and fibrosis regression-D3 time points. Inverse correlation of KC frequency with CD45neg SSChi population at Fibrosis Regression-D3 **(G)**. Correlation of CD45neg SSChi cells frequency with necrosis scoring at fibrosis regression-D3 time point **(H)**. Symbols represent values from individual mice. Group mean values are presented. Statistics: For necrosis and Ishak grade scorings, groups were compared using Chi-square test (n=18-48 mice/group) **(C)**. Two-way ANOVA in **(E)** and **(F)** Pearson’s correlation test in **(G**, **H)** *, p<0.05 ***, p<0.001, ****, p<0.0001.

To extend our observations to chronic liver damage wild-type and Trem-2 KO mice were exposed to carbon tetrachloride (CCl4) treatment for 4 weeks and analyzed on day 1 (D1) and day 3 (D3) after treatment corresponding to established liver fibrosis and fibrosis regression time points, respectively (**Figure 3A**). At D1 Trem-2 KO mice showed a stronger fibrotic phenotype, with increased hepatocyte necrosis and fiber deposition (**Figures 3B–D**). Strikingly, fibrosis resolution response was compromised in Trem-2 KO mice by D3 as indicated by persistence of necrosis and collagen deposition that contrasted with nearly complete necrosis clearance and significant fibrosis regression in wild-type mice (**Figures 3B–D**).

Accordingly, analysis of macrophage dynamics revealed that recruitment of macrophages at D1 was not affected in Trem-2 KO mice but at D3 KCs replenishment was impaired and non-hematopoietic CD45^neg^ SSC^hi^ cells were notoriously overrepresented (**Figures 3E, F**). Additionally, serum levels of CD26 activity tend to be higher at D3 in Trem-2 KO mice (**Supplementary Figure 2B**), strengthening the notion that KCs recovery was delayed in these mice. Similar to the acute model, CD45^neg^ SSC^hi^ cells accumulation at D3 was inversely correlated with KC proportions (**Figure 3G**) and associated with the persistence of liver necrosis (**Figure 3H**). Furthermore, genetic expression of TNF-α in non-parenchymal cells (NPCs) correlated with the proportions of CD45^neg^ SSC^hi^ cell population (**Supplementary Figure 4**). These data reinforce that ablation of Trem-2 impacts the dynamics of macrophage populations favoring a pro-inflammatory milieu and accumulation of CD45^neg^ SSC^hi^ cells that are in turn associated with delayed resolution of tissue damage.

### Trem-2 Is Upregulated in Transition Macrophages Promoting Acquisition of Resident-Like Phenotype

**Figure 4:**
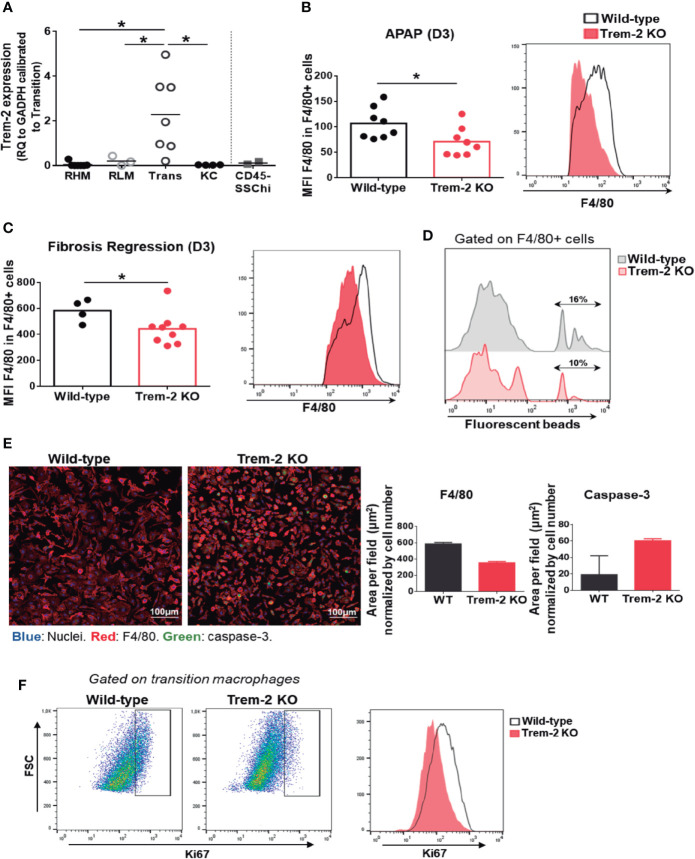
Trem-2 is implicated in acquisition of liver transition macrophages phenotype. Trem-2 gene expression was evaluated by qPCR in sort-purified macrophage populations and in non-hematopoietic cell population, CD45neg SSChi in wild-type mice and represented as fold change to transition macrophages **(A)**. F4/80 mean fluorescence intensity (MFI) among F4/80int/high non-parenchymal cells from wild-type and Trem-2 KO mice at APAP-D3 **(B)** and fibrosis regression-D3 **(C)**. Percent of F4/80int/high non-parenchymal cells containing fluorescent beads in wild-type and Trem-2 KO at APAP-D3 1 h after i.v. injection of 50x106 2μm Yellow-Green fluorescent microspheres **(D)**. Immunofluorescence of sort-purified APAP-D3 transition macrophages from wild-type and Trem-2 KO mice after 3 days of culture (Nuclei (blue), F4/80 (red), caspase-3 (green). Merged images are shown and quantification of F4/80 and caspase-3 stained area normalized by nuclei is plotted **(E)**. Representative flow cytometry plots showing Ki67 staining in APAP-D3 transition macrophages from wild-type and Trem-2 KO mice **(F)**. Statistics: One-way ANOVA and Tukey’s correction in **(A)** Mann Whitney test in **(B**, **C)** *, p<0.05.

Trem-2 RNA expression was quantified in sort-purified macrophage populations and non-hematopoietic CD45^neg^ SSC^hi^ cells from wild-type mice 3 days after APAP treatment (D3) (**Supplementary Figure 5A**). Remarkably, Trem-2 expression was upregulated in transition macrophages and almost undetectable in the other macrophage populations (**Figure 4A**). This strongly suggests that expression of Trem-2 in the transition macrophage population promotes adequate dynamics of KCs replenishment. Furthermore, Trem-2 was not expressed in CD45^neg^ SSC^hi^ cells (**Figure 4A**) suggesting that their accumulation in Trem-2 KO mice may result from abnormal macrophage responses.

Analysis of F4/80 surface expression revealed that Ly6c^-^ macrophages from Trem-2 KO mice present decreased surface expression of F4/80 molecule upon acute (**Figure 4B**) and chronic (**Figure 4C**) injury. An *in vivo* phagocytosis functional assay with fluorescent beads at D3 after APAP treatment show that Trem-2 KO macrophages have decreased ability to phagocytose compared to wild-type (**Figure 4D**). Furthermore, we found that Trem-2 KO transition macrophages in culture maintain a rounder shape as assessed by the total F4/80 area (**Figure 4E**), while wild-type macrophages acquire a typical KC-like morphology. This suggests that Trem-2 is involved in the phenotype switch from transition to resident-like macrophage. Quantification of caspase-3 expression in culture and ki67 *ex vivo* staining in Trem-2 KO transition macrophages showed increased apoptosis and decreased proliferation (**Figures 4E, F**). These results indicate that ablation of Trem-2 impairs KCs replenishment possibly by controlling cell survival and therefore allowing the transition into KCs.

### Trem-2 Increases Resilience to Oxidative Stress and Swift Shutdown of Pro-Inflammatory Program in Transition Macrophages

**Figure 5:**
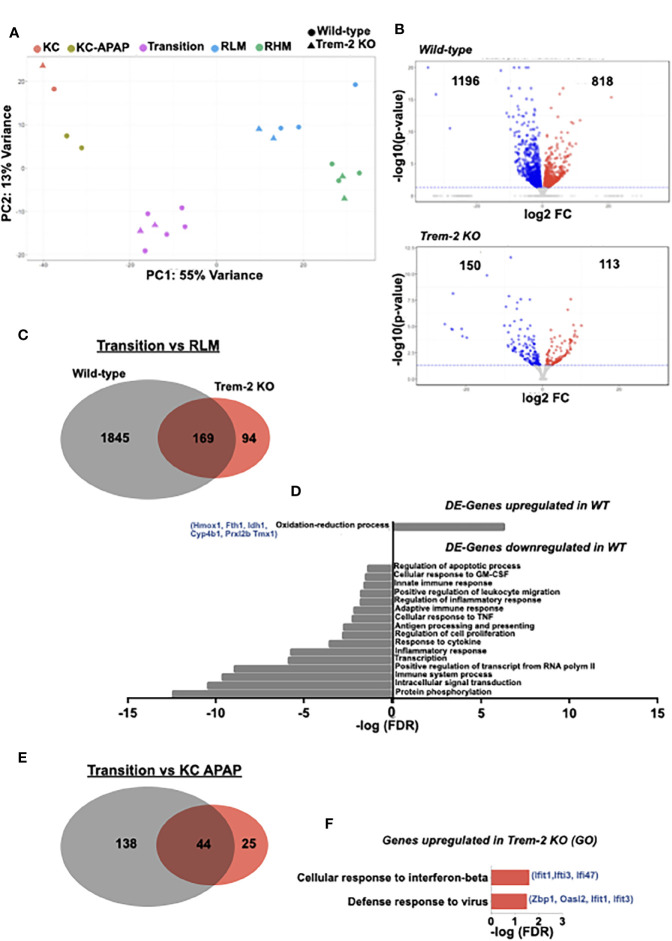
Transcriptomic profiling identifies a Trem-2 dependent program in transition macrophages. Principal component analysis (PCA) of transcriptomic data representing clustering of different sort-purified macrophage populations (represented by colors) from wild-type (circles) and Trem-2 KO mice (triangles) **(A)**. Volcano plots representing differential expressed (DE) genes (q<0.05) between transition and recruited-like macrophages (RLM) in wild-type and Trem-2 KO mice. Red dots represent upregulated genes (LogFC>0), while blue dots represent downregulated genes (LogFC<0) **(B)**. Venn diagram representing DE-genes between transition and RLM, which are common to wild-type and Trem-2 KO mice (middle), exclusively detected in wild-type (left) or in Trem-2 KO (right) **(C)**. Gene Ontology (GO) enrichment analysis in the “Biological Process” category for DE-genes upregulated or downregulated in transition versus RLM in wild-type mice **(D)**. Venn diagram representing DE-genes upregulated in transition macrophages versus Kupffer cells (KC APAP-D3) in wild-type and Trem-2 KO mice **(E)**. Gene Ontology (GO) enrichment analysis in the “Biological Process” category for DE-genes exclusively upregulated in transition macrophages of Trem-2 KO mice **(F)**. GO terms over-represented for FDR (Bejamini)<0.05.

To better discern the functional role of Trem-2 in macrophage phenotypic shifts we performed transcriptomic analysis in sort-purified macrophage populations of wild-type and Trem-2 KO mice (**Supplementary Figure 4A**) at D3 after APAP treatment. Hierarchical algorithms and principal component analysis (PCA) clustered the different macrophage populations and clearly showed that the transcriptional profiles of RHM are in the vicinity of RLM and that transitional macrophages are closer to KCs (**Figure 5A** and **Supplementary Figure 5A**). This is in agreement with accepted notions that recruited macrophages (RHM) home the liver as inflammatory macrophages and loose Ly6c expression subsequently giving rise to transition macrophages and KCs (1, 29).

On the other hand, wild-type and Trem-2 samples were clustered within each macrophage population (**Figure 5A**), suggesting that Trem-2 does not have a major impact in the global macrophage transcriptional programs. Given that Trem-2 affects the dynamical switching of transitional macrophage populations we performed a detailed analysis of differentially expressed (DE) genes comparing RLM and transition macrophages. We found that the transcriptional shift was more prominent in wild-type mice than in Trem-2 KO mice (**Figures 5B, C**). Interestingly, this shift encompassed the upregulation of genes associated to oxidation-reduction processes and downregulation of genes associated with inflammatory responses (**Figure 5D**), a pattern that was not observed in Trem-2 KO cells. We measured by qPCR the expression of two of these upregulated genes (Hmox1 and Fth1) in sorted transition macrophages confirming that activation of oxidative stress response mechanisms are blunted in Trem-2 KO transition macrophages (**Supplementary Figure 6A**). This analysis suggests that the transition macrophage transcriptional program was not fully acquired in Trem-2 KO cells.

Furthermore, comparison of transition macrophages and replenished KCs again showed that the transcriptional program switch is considerably less prominent in Trem-2 KO mice (**Figure 5E**). Gene ontology analysis revealed that genes specifically upregulated in transition macrophages from Trem-2 KO mice are associated to interferon-beta response, suggesting that these cells sustained a pro-inflammatory profile (**Figure 5F**). In addition, we analyzed the transcriptional switch between recruited macrophage populations comparing RHM to RLM and found a similar change extent in wild-type and Trem-2 KO mice (**Supplementary Figure 6B**). Moreover, gene ontology analysis showed that irrespective of Trem-2 expression, RLM have a transcriptional profile of increased proliferative capacity (**Supplementary Figure 6C**). Taken together these results show that Trem-2 expression in transition macrophages is key to shutdown the pro-inflammatory transcriptional program and increase resilience to oxidative stress during acquisition of resident macrophage functions.

### CD45^neg^ SSC^hi^ Cells Present an Endothelial Lineage Transcriptomic Profile

**Figure 6:**
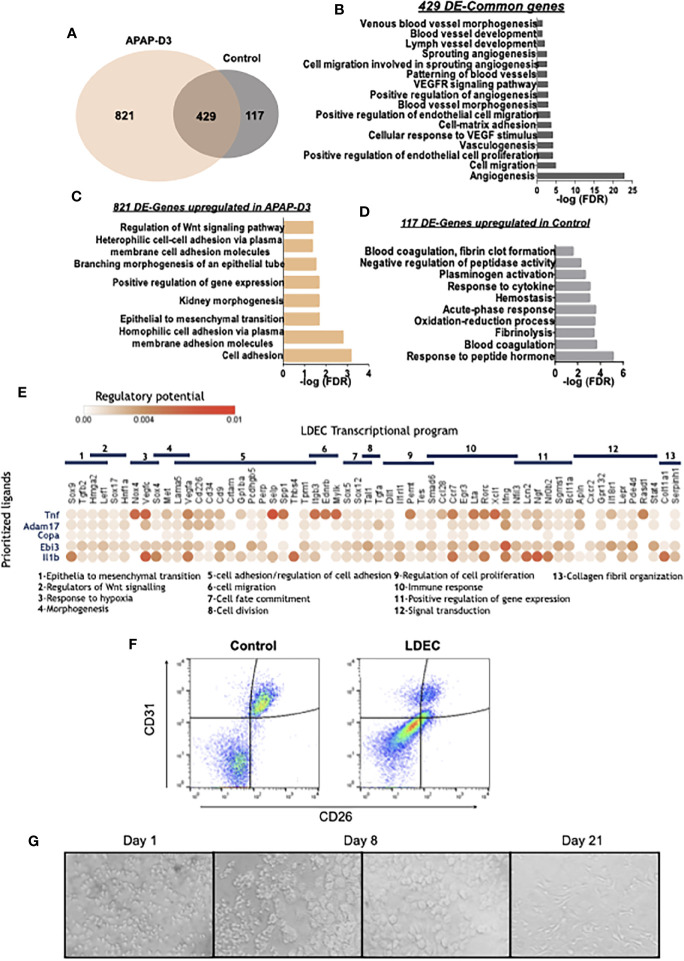
LDECs identified among the CD45neg SSChi population by transcriptomic and phenotypic analysis and their interaction with transition macrophages. Venn diagram representing differentially expressed genes (DE-genes) upregulated in CD45neg SSChi population from APAP-D3 and untreated control mice. Kupffer cells (KC APAP-D3) transcriptomic data was used as a reference for DE-genes comparison **(A)**. Gene Ontology (GO) enrichment analysis in the ‘Biological Process’ category for common DE upregulated genes in APAP-D3 and control compared to KCs **(B)** for DE upregulated genes exclusively in APAP-D3 **(C)** and genes upregulated exclusively control mice **(D)**. GO terms over-represented for FDR (Bejamini)<0.05. Dot plot of ligand-receptor interactions between transition macrophages (y axis) and LDECs populations (x axis). Circle color denotes average strength of the regulatory potential between sender and receiver cells. LDECs associated gene ontology terms are identified **(E)**. Flow cytometry plots showing double staining for CD31 and CD26 in control and APAP-D3 (LDECs) CD45neg SSChi cells **(F)**. Sort-purified LDECs cultured in M-CSF supplemented medium at 1, 8 and 21 days **(G)**.

We next sought to discern the transcriptional program of the non-hematopoietic CD45^neg^ SSC^hi^ cells that accumulate in the liver in response to tissue injury and correlate with reduced liver KCs and persistent liver damage (**Figures 2D, E** and **Figures 3G, H**). The transcriptomic profiles of sorted CD45^neg^ SSC^hi^ cells in APAP-treated (D3) and untreated mice (**Supplementary Figure 4B**) were closely related when using KCs as a reference population (**Figure 6A** and **Supplementary Figure 5B**). Gene ontology analysis performed for the DE-genes common to control and APAP-D3 CD45^neg^ SSC^hi^ cells showed striking enrichment in terms related to endothelial cell identity and function (**Figure 6B**). In addition, DE-genes upregulated in CD45^neg^ SSC^hi^ cells of APAP–D3 treated mice revealed enrichment in pathways related to epithelial to mesenchymal transition and to regulation of Wnt signaling pathway (**Figure 6C**). On the other hand, DE-genes up-regulated in CD45^neg^ SSC^hi^ cells from control mice revealed enrichment in functional pathways involved in blood coagulation, hemostasis and fibrinolysis, typical of endothelial cells (**Figure 6D**). These transcriptomic data clearly identified the CD45^neg^ SSC^hi^ population accumulating in the damaged liver as belonging to the endothelial cell lineage leading us to operationally name these cells as Liver Damage-associated Endothelial Cells (LDECs). In addition, the ontology analysis suggests that LDECs are undergoing endothelial de-differentiation.

We used *in silico* analysis to infer interactions between transition macrophages and LDECs during liver tissue repair. Niche Net (30), an algorithm that predicts ligand-receptor interactions by combining transcriptome data of interacting cells revealed that TNF-α, ADAM17, Copa, Ebi3 and Il1β were the top 5 transition macrophages ligands connected with LDECs transcriptional profile, irrespective of the mice genotype (**Figure 6E**), This supports that a sustained pro-inflammatory profile during macrophage phenotypic transitions, as observed in Trem-2 KO mice, promotes LDECs accumulation.

### LDECs Proliferate and Differentiate *In Vitro*

We characterized LDECs by flow cytometry using liver endothelial cell surface makers, namely CD31 and CD26. Strikingly, LDECs express lower levels of these markers as compared to endothelial cells from control mice (**Figure 6F**). This is line with previous reports showing that endothelial cells undergoing endothelial to mesenchymal transition downregulate endothelial specific markers such as CD31 (31). We next explored the ability of sort-purified LDECs to differentiate *in vitro* in presence of macrophage-colony stimulating factor (M-CSF), a growth factor able to promote angiogenesis (32). LEDCs were very small and granulous until day 8 when larger sized, round-shaped cells emerged and expanded in the culture, eventually developing typical endothelial morphology by 21 days (**Figure 6G**). These results reveal that LDECs are at a particular activation state characterized by down-modulation of endothelial cell markers and ability to differentiate into morphologically distinct cells, indicative of their precursor potential.

## Discussion

This work revealed that Trem-2 controls the replenishment of liver macrophage populations after acute and chronic hepatotoxic damage and is a critical determinant of swift tissue repair responses conditioning the emergence of endothelial lineage cells during regeneration. Recent reports proposed that lack of Trem-2 expression in non-parenchymal cells contributes to increased fibrosis in Trem-2 KO mice submitted to CCl4 treatment, to increased liver inflammation after acute APAP treatment (24) and to increased susceptibility to hepatocarcinogenesis (33). We noted that Trem-2 KO mice did not show significantly higher intensity of liver damage during acute injury (APAP-D1) but showed sustained tissue damage throughout the tissue repair phase (APAP-D3). Likewise, Trem-2 KO mice presented slightly heightened necrosis during the inflammatory phase of chronic liver injury (CCl4-D1) and a marked delay in subsequent resolution of tissue necrosis and fibrosis (CCl4-D3). While Trem-2 may influence the initial hepatic inflammatory and fibrotic reactions (24), here we focused on its impact in liver macrophages responses during recovery from drug-induced damage.

Macrophage phenotypic plasticity is well illustrated in the responses to liver tissue damage (8, 9). In particular, we identified a transition macrophage population that expresses Trem-2 in high levels and predominates during the recovery phase from acute and chronic damage. These cells are derived from circulating monocytes and both transcriptional and phenotypic profiling placed them as the immediate source of resident-like macrophages (Kupffer cells) in the recovered liver. These observations strongly suggest that Trem-2 signaling plays a key role in the dynamics and efficiency of this phenotypic transition, thus explaining the delayed replenishment of the KCs compartment in Trem-2 KO mice. Noteworthy, recent reports have shown that replenishment of the Kupffer cell niche occurs upon engraftment of circulating monocytes into the perisinusoidal space, which is dependent of coordinate interactions between hepatocytes, stellate cells and endothelial cells in the liver (34). These interactions induce a particular transcriptional program and epigenetic changes, responsible for the induction and maintenance of Kupffer cell identity (35). Also, a scar-associated Trem2+CD9+ subpopulation of macrophages was identified in cirrhotic human livers (36). Interestingly, a recent report has shown that in a mouse model of NASH, Kupffer cells are lost mostly likely by apoptosis which induced Trem-2 expression and replenishment of the KC compartment by monocyte-derived macrophages (37).

In addition, a Trem-2 dependent transcriptional program has been associated with emergence of restorative macrophage populations in the context of brain tissue degeneration and adipose tissue inflammation (21, 22). Similarly, liver transition macrophages show upregulation of genes associated with this Trem-2 dependent transcriptional signature (**Supplementary Figure 7**).

Taken together, our results contribute to the identification of Trem-2 as a critical determinant of macrophage plasticity instrumental for a swift recovery from tissue damage.

Acquisition of the transition phenotype in wild-type macrophages includes down-modulation of pro-inflammatory genes and up-regulation of genes involved in oxidative stress responses, not observed in Trem-2 KO macrophages. Redox regulation is critical to cellular stress control mechanisms (38). Accordingly, transition macrophages from Trem-2 KO mice showed decreased survival and proliferative capacities. These observations are in line with reports of reduced survival of Trem-2 KO microglia cells during neurodegenerative processes (17, 39). Furthermore, reactive oxygen species (ROS) were increased in Trem-2 KO bone-marrow derived macrophages and hepatic lipid peroxides were increased during liver damage in Trem-2 KO mice (24). Together, these findings indicate that transition macrophages may play a relevant role in ROS clearance in damaged liver and that Trem-2 expression is likely fundamental for transition macrophage survival and proliferation, therefore allowing their acquisition of KCs phenotype.

Remarkably, ablation of Trem-2 leads to increased accumulation of a non-hematopoietic population that we identified as Liver Damage Endothelial Cells (LDECs). This population appeared during tissue repair phases and its accumulation correlated with the severity of tissue damage. It has been recently proposed that vascular endothelial stem cells residing in the liver are activated upon acute liver injury and act as angiogenesis-initiating cells showing remarkable vascular regenerative capacity (40). On the other hand, a specific subset of liver sinusoidal endothelial cells was found to sustain liver regeneration after hepatectomy by releasing angiocrine trophogens (41). Nevertheless, the development and rules of engagement of endothelial progenitors in liver angiogenic repair responses remain unclear.

LDECs transcriptional profile denotes a differential functional activation status as compared to endothelial cells from untreated livers. Gene ontology analysis revealed that LDECs are involved in biological processes related to epithelial to mesenchymal transition (**Figure 6C**). A similar process designated endothelial to mesenchymal transition (EndMT) has been reported to play important roles in pathogenesis of many diseases (42) as well as in regenerative processes (31). EndMT promotes cell de-differentiation, consequently giving rise to mesenchymal stem cells with the ability to differentiate into new cell types (43). These cells were shown to differentiate into endothelial cells that contribute to neovascularization (42). Interestingly, M-CSF, an essential regulator of macrophage development, induced marked LDECs morphological changes *in vitro*, highlighting their de-differentiated state and intrinsic proliferative and differentiation potential.

*In silico* analysis of predicted ligand-receptor suggests an interaction between transition macrophages and LDECs, where inflammatory mediators secreted by macrophages activate a transcriptional profile on endothelial cells. Of note, ligands such as TNF-α and Il1β showed established correlation with transcription of Sox9, Selp and Ccr7, which are expressed by LDECs. It is well accepted that TNF-α and Il1β are able to activate endothelial cells and promote angiogenesis (44, 45) as well as potentiate leukocyte transmigration into inflamed tissues (46). Noteworthy, a recent report used *in silico* analysis to highlight liver macrophage-endothelial cell interactions in the context of cirrhosis. The authors describe a particular scar associated macrophage subpopulation expressing Trem2 as well as endothelial populations restricted to the fibrotic niche (36). These observations corroborate our findings that, in damaged liver, macrophages and endothelial cell populations are interlinked and may represent a hallmark of liver regenerative responses. Nevertheless, the precise mechanism mediating this interaction warrants further investigation.

In sum, this work describes Trem-2 as a promotor of macrophage phenotypic switching during tissue repair, tuning down the recruited macrophage inflammatory profile, enhancing oxidation-reduction responses and allowing KCs replenishment. In parallel, we identified an endothelial cell population (LDECs) that emerges during tissue repair with a distinct transcriptional profile and phenotypic features of endothelial de-differentiation.

## Data Availability Statement

The datasets presented in this study can be found in online repositories. The names of the repository/repositories and accession number(s) can be found in the article/**Supplementary Material**.

## Ethics Statement

The animal study was reviewed and approved by Comissão de Ética do Instituto Gulbenkian de Ciência.

## Author Contributions

IC designed and performed experiments, and drafted the paper. ND designed and performed experiments, and drafted the paper. AB analyzed the RNASeq data. MPM conceived the project and discussed results. CP-G conceived the project, supervised the work, and drafted the paper. All authors contributed to the article and approved the submitted version.

## Funding

This work was developed with the support of the research infrastructure Congento, project LISBOA-01-0145-FEDER-022170, co-financed by Lisboa Regional Operational Programme (Lisboa 2020), under the Portugal 2020 Partnership Agreement, through the European Regional Development Fund (ERDF), and FCT – “Fundação para a Ciência e a Tecnologia” (Portugal). This work was partially supported by ONEIDA project (LISBOA-01-0145-FEDER-016417) co-funded by FEEI – “Fundos Europeus Estruturais e de Investimento” from “Programa Operacional Regional Lisboa 2020” and by national funds from FCT through grants PTDC/BIM-MET/2115/2014, PTDC/BIM-MET/4265/2014 and iNOVA4Health (UID/Multi/04462/2013). IC was supported by a FCT fellowship PD/BD/105997/2014.

## Conflict of Interest

The authors declare that the research was conducted in the absence of any commercial or financial relationships that could be construed as a potential conflict of interest.

## Supplementary Material

Supplementary Figure 1 Transition macrophages and Kupffer cells show different expression of Tim4 and Clec4f. Flow cytometry analysis of liver non-parenchymal cells (NPCs) from wild-type and Trem-2 KO mice, untreated and at D3 post APAP injury. Macrophage populations were identified within CD45+ cells. Resident macrophage makers, Tim4 and Clec4f, were used to identify Kupffer cells. Transition macrophages and Kupffer cells were identified using CD11b and F4/80 within Tim4+Clec4f+ gate and within Tim4-Clec4f- gate.

Supplementary Figure 2 Serum CD26 activity during recovery from acute liver damage and fibrosis regression. CD26 activity represented in arbitrary units (A.U.) was measured in the serum of wild-type and Trem-2 KO mice, during recovery from APAP (D3) **(A)** and during fibrosis regression (D3) induced by CCl4 **(B)** or in mice left untreated (Ctrl). Symbols represent individual mice. One-way ANOVA *, p<0.05 ***, p<0.001.

Supplementary Figure 3 Recruited monocyte tracking in liver macrophage populations following acute liver damage. Monocytes from B6.Actin-GFP mice were intravenously injected 12 hours after APAP treatment in wild-type mice and non-parenchymal cells were isolated at APAP-D3. Flow cytometry histograms show enrichment in GFP+ cells compared to non-transferred mice in different macrophage populations: RLM, Transition and Kupffer cells.

Supplementary Figure 4 Accumulation of CD45neg SSChi cells correlates with expression of pro-inflammatory cytokines in the liver. Positive correlation between frequency of CD45neg SSChi population and TNFα gene expression in liver non-parenchymal cells during recovery from chronic injury (CCl4-D3). Statistics: Pearson’s correlation test.

Supplementary Figure 5 FACsort-purified macrophage populations and CD45neg SSChi cells for transcriptomic analysis. Wild-type and Trem-2 KO mice received a single intraperitoneal injection of APAP and macrophage populations and CD45neg SSChi cells were sorted at APAP-D3 and in control mice. Macrophage populations were identified using CD45, Ly6c, CD11b and F4/80 markers and the purity of each population after sorting is shown **(A)**. CD45neg SSChi population was identified using CD45 marker in APAP-D3 and in control mice and the purity after sorting is shown **(B)**. Each sample for transcriptomic analysis was obtained from pools of 4 mice.

Supplementary Figure 6 Heatmaps of DE-genes representing hierarchical clustering of macrophage and CD45neg SSChi cell populations. Heatmap represents 100 KCs associated genes42 for different macrophage populations **(A)**. Heatmap showing differentially expressed (DE) genes in CD45neg SSChi cells using KCs as reference: 821 genes upregulated in APAP-D3, 117 upregulated in control and 428 common to APAP-D3 and control as identified in **Figure 2**.6A **(B)**.

Supplementary Figure 7 Gene expression and transcriptomic analysis of macrophage populations. Hmox1 and Fth1 gene expression was evaluated by qPCR in sort-purified KCs, transition and RLM populations from wild-type and Trem-2 KO mice at APAP-D3 **(A)**. Volcano plots representing differential expressed (DE) genes (q<0.05) between RLM and RHM in wild-type and Trem-2 KO mice. Red dots represent upregulated genes with LogFC>0, while blue dots represent downregulated genes with LogFC<0 significant for q<0.05 **(B)**. Venn diagram representing DE-genes between RLM and RHM which are common to wild-type and Trem-2 KO (middle), exclusive for wild-type (left) or Trem-2 KO (right). Gene Ontology (GO) enrichment analysis in the ‘Biological Process’ category for DE-genes in wild-type mice and Trem-2 KO mice **(C)**. Heatmap illustrating genes regulated by Trem-2 in transition macrophages. Log fold change (logFC) of genes previously associated to Trem-2 transcriptional signature (21, 22). Heatmap represents DE-genes upregulated in transition versus RLM at APAP-D3 in wild-type and Trem-2 KO mice
