# Supplementary material for: Trem-2 Promotes Emergence of Restorative Macrophages and Endothelial Cells During Recovery From Hepatic Tissue Damage
Source: Front Immunol. 2021 Feb 8;11:616044. doi: 10.3389/fimmu.2020.616044 (PMC7897679; doi:10.3389/fimmu.2020.616044)
Supplement: Supplementary file 1 [file Table_1.docx]

**Supplementary Materials and Methods**

***CD26/DPP-4 enzymatic activity***

Serum from total blood was measured for DPP-4 activity using a fluorimetric assay with 200 uM of the substrate H-Gly-Pro-AMC HBr (#I-1225, Bachem, Bubendorf, Switzerland). Measurements at excitation/emission 360/460 nm were taken every 5 minutes during one hour and the slope of fluorescence was used as measure of cleaved substrate by CD26/DPP-4, reported as arbitrary units.

***Monocyte transfers***

To track recruited macrophages in the liver we isolated monocytes from the spleen of B6.Actin-GFP mice as previously described (1). Briefly, spleens were collected, smashed and red blood cells lysed. Cells were firstly stained with biotinylated anti-TCR-β, anti-B220 and anti-NK1.1 antibodies and depleted with anti-biotin microbeads using MACS LD columns (MACS- Miltenyl Biotec). GFP-labeled monocytes, identified as Mac1^+^ and Ly6c^+^ by flow cytometry were counted and 2.5x10^6^ cells were injected intravenously in wild-type recipients. (See antibodies table below).

***Image Analysis***

Quantification of F4/80 fluorescence was obtained by thresholding each stitched tile using the Mean Auto-Threshold algorithm of FIJI software package (2) and measuring the total area of F4/80 normalized by the number of nuclei to control for cell confluency. Total number of nuclei was determined by thresholding the DAPI channel using the Otsu Auto-Threshold algorithm and counting them through FIJI particle analyzer functions.

Similar methods were employed to quantify Caspase-3 fluorescence, applying the Moments Auto-Threshold and dividing total area by number of nuclei for normalization. Total number of nuclei was obtained by making each stitched tile binary in the DAPI channel, applying the Watershed function, and recording total Count.

**RNA Sequencing analysis**

Quality Assessment and Alignment. Prior to alignment, quality of the sequences was assessed using FASTQC (3) and MultiQC (4). Alignment was performed against the *Mus musculus* genome version 95, with the annotation file for the genome version 95, both obtained through the Ensembl website and using STAR (5), with default parameters and with the option of *GeneCounts*.

Data Analysis***.*** The files obtained from *GeneCounts* option were imported to R, taking into account the strandness inherent to the sequencing protocol. Downstream analysis was performed using DESeq2 (version 1.22.2) (6). Data from raw counts used to create the Principal Component Analysis plot and Heatmaps were normalized through a Variance Stabilizing Transformation (VST) (7). The log2FC provided by the standard DESeq2 model was shrunk using the ‘ashr’ option (8). Gene Information was obtained using the package *org.Mm.eg.db*. For the purposes of this study, genes were considered differentially expressed when the p-value, adjusted using false discovery rate (FDR), was below 0.05. Log2FC tables of differentially expressed genes (DE-genes) are available at https://figshare.com/s/3684f039abc8c96313a3. Gene ontology analysis was performed using DAVID Bioinformatics Resources 6.8 webtool (https://david.ncifcrf.gov/) and enriched terms considered for FDR<0.05 using Benjamini Hochberg method.

To perform ligand to gene prediction we used the NicheNet algorithm (9). This analysis allows linking the activity of a ‘sender cell’, through its ligands, to a transcriptional program of a ‘receiver cell’. In our dataset we determined Transition macrophages as the sender cells and LDECs as the receivers. To sort the genes expressed at medium and high level in both cell types we followed a TPM-based approach, considering only the genes which had a mean log2 TPM values higher than 3, a criterion adapted from (10). In order to transform the gene counts into TPM values, we used the total gene counts and the gene length obtained using the biomaRt package v.2.38.0 (11) and the corresponding Ensembl version. To apply NicheNet pipeline we used available information (ligand-to-target matrix and network) (12) and a transcriptional program defined by us. These were filtered to include only the ligands expressed by the sender cells as well as the receptors expressed in the receiver cells. To determine the most relevant ligands explaining the downstream transcriptional signature, we used the Pearson Correlation Coefficient values, as recommended by the algorithm’s authors (12). Regarding network analysis, we performed several iterations, modifying the number of ligands and the size of the considered database.

Data and Code Availability

The RNA-seq datasets and scripts generated during this study are available on GitHub: https://github.com/andrebolerbarros/RNASeq_icoelho_etal_2020/.

**Antibodies**

| **Immunohistochemistry/ Immunofluorescence** |  |  |
| --- | --- | --- |
| **Antibody** | **Supplier** | **Clone** |
| Rat anti-F4/80 | BioRad | Cl:A3-1 |
| Rabbit anti-Caspase-3 | Abcam | polyclonal |
| Goat anti-rat IgG Alexa Fluor 647 | ThermoFisher | polyclonal |
| Goat anti-rabbit IgG Alexa Fluor 568 | ThermoFisher | polyclonal |
| **Flow Cytometry** |  |  |
| **Antibody** | **Supplier** | **Clone** |
| Anti-mouse-Fc-block/CD16/32 | IGC (antibody facility) | 2.4G2 |
| Anti-mouse PE CD45 | BioLegend | 104.2 |
| Anti-mouse Alexa-700 F4/80 | BioLegend | BM8 |
| Anti-mouse efluor450 Ly6c | BioLegend | HK1.4 |
| Anti-mouse Brilliant Violet-785-CD11b/Mac-1 | eBiosciences | M1/70 |
| Anti-mouse Alexa-488 Ki67 | BD Pharmigen | B56 |
| Anti-mouse Fitc CD26 | BioLegend | H194-112 |
| Anti-mouse APC CD31 | BD Pharmigen | MEC 13.3 |
| Anti-mouse biotinylated TCR beta | IGC (antibody facility) | H57-597 |
| Anti-mouse biotnylated B220 | IGC (antibody facility) | RA3-6B2 |
| Anti-mouse biotinylated NK1.1 | IGC (antibody facility) | PK136 |

**References**

1. Girgis NM, Gundra UM, Ward LN, Cabrera M, Frevert U, Loke P. Ly6Chigh Monocytes Become Alternatively Activated Macrophages in Schistosome Granulomas with Help from CD4+ Cells. *PLoS Pathog* (2014) **10**: doi:10.1371/journal.ppat.1004080

2. Schindelin J, Arganda-Carreras I, Frise E, Kaynig V, Longair M, Pietzsch T, Preibisch S, Rueden C, Saalfeld S, Schmid B, et al. Fiji: An open-source platform for biological-image analysis. *Nat Methods* (2012) **9**:676–682. doi:10.1038/nmeth.2019

3. Andrews S. FastQC: a quality control tool for high throughput sequence data. *Available: https://www.bioinformatics.babraham.ac.uk/projects/fastqc/* (2010) Available at: http://www.bioinformatics.babraham.ac.uk/projects/fastqc

4. Ewels P, Magnusson M, Lundin S, Käller M. MultiQC: Summarize analysis results for multiple tools and samples in a single report. *Bioinformatics* (2016) **32**:3047–3048. doi:10.1093/bioinformatics/btw354

5. Dobin A, Davis CA, Schlesinger F, Drenkow J, Zaleski C, Jha S, Batut P, Chaisson M, Gingeras TR. STAR: Ultrafast universal RNA-seq aligner. *Bioinformatics* (2013) **29**:15–21. doi:10.1093/bioinformatics/bts635

6. Love MI, Huber W, Anders S. Moderated estimation of fold change and dispersion for RNA-seq data with DESeq2. *Genome Biol* (2014) **15**:1–21. doi:10.1186/s13059-014-0550-8

7. Anders S, Huber W. Differential expression analysis for sequence count data. *Genome Biol* (2010) **11**:R106. doi:10.1186/gb-2010-11-10-r106

8. Stephens M. False discovery rates: A new deal. *Biostatistics* (2017) **18**:275–294. doi:10.1093/biostatistics/kxw041

9. Browaeys R, Saelens W, Saeys Y. NicheNet: modeling intercellular communication by linking ligands to target genes. *Nat Methods* (2019) doi:10.1038/s41592-019-0667-5

10. Puram S V., Tirosh I, Parikh AS, Patel AP, Yizhak K, Gillespie S, Rodman C, Luo CL, Mroz EA, Emerick KS, et al. Single-Cell Transcriptomic Analysis of Primary and Metastatic Tumor Ecosystems in Head and Neck Cancer. *Cell* (2017) **171**:1611-1624.e24. doi:10.1016/j.cell.2017.10.044

11. Durinck S, Spellman PT, Birney E, Huber W. Mapping identifiers for the integration of genomic datasets with the R/ Bioconductor package biomaRt. *Nat Protoc* (2009) **4**:1184–1191. doi:10.1038/nprot.2009.97

12. Browaeys R, Saelens W, Saeys Y. Development, evaluation and application of NicheNet: datasets [Data set]. *Zenodo* (2019) Available at: http://dx.doi.org/10.5281/zenodo.3260758
