# Supplementary figures and images for: Trem-2 Promotes Emergence of Restorative Macrophages and Endothelial Cells During Recovery From Hepatic Tissue Damage
Source: Front Immunol. 2021 Feb 8;11:616044. doi: 10.3389/fimmu.2020.616044 (PMC7897679; doi:10.3389/fimmu.2020.616044)

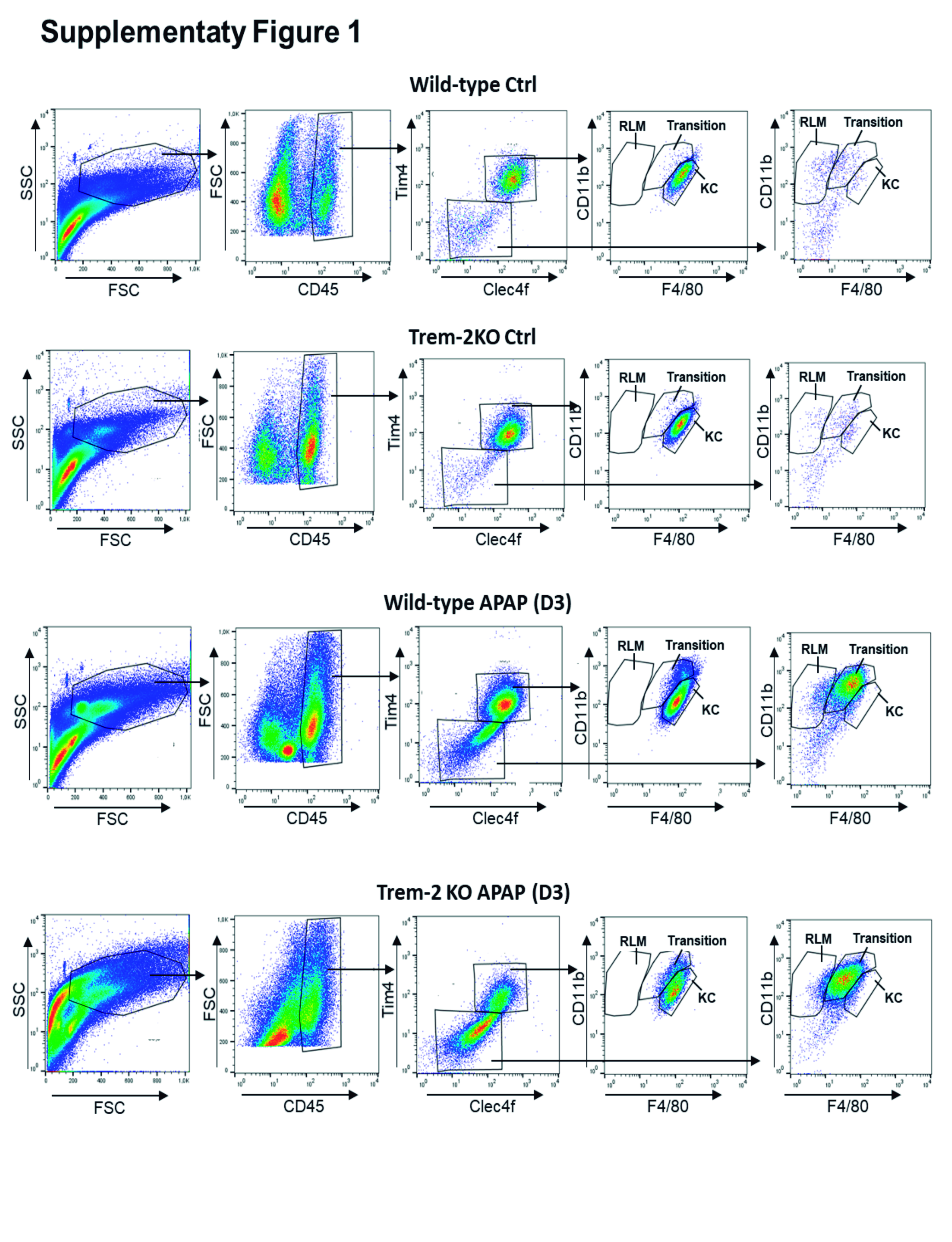

Supplement: Supplementary Figure 1 — Transition macrophages and Kupffer cells show different expression of Tim4 and Clec4f. Flow cytometry analysis of liver non-parenchymal cells (NPCs) from wild-type and Trem-2 KO mice, untreated and at D3 post APAP injury. Macrophage populations were identified within CD45+ cells. Resident macrophage makers, Tim4 and Clec4f, were used to identify Kupffer cells. Transition macrophages and Kupffer cells were identified using CD11b and F4/80 within Tim4+Clec4f+ gate and within Tim4-Clec4f- gate. [file Image_1.tif]

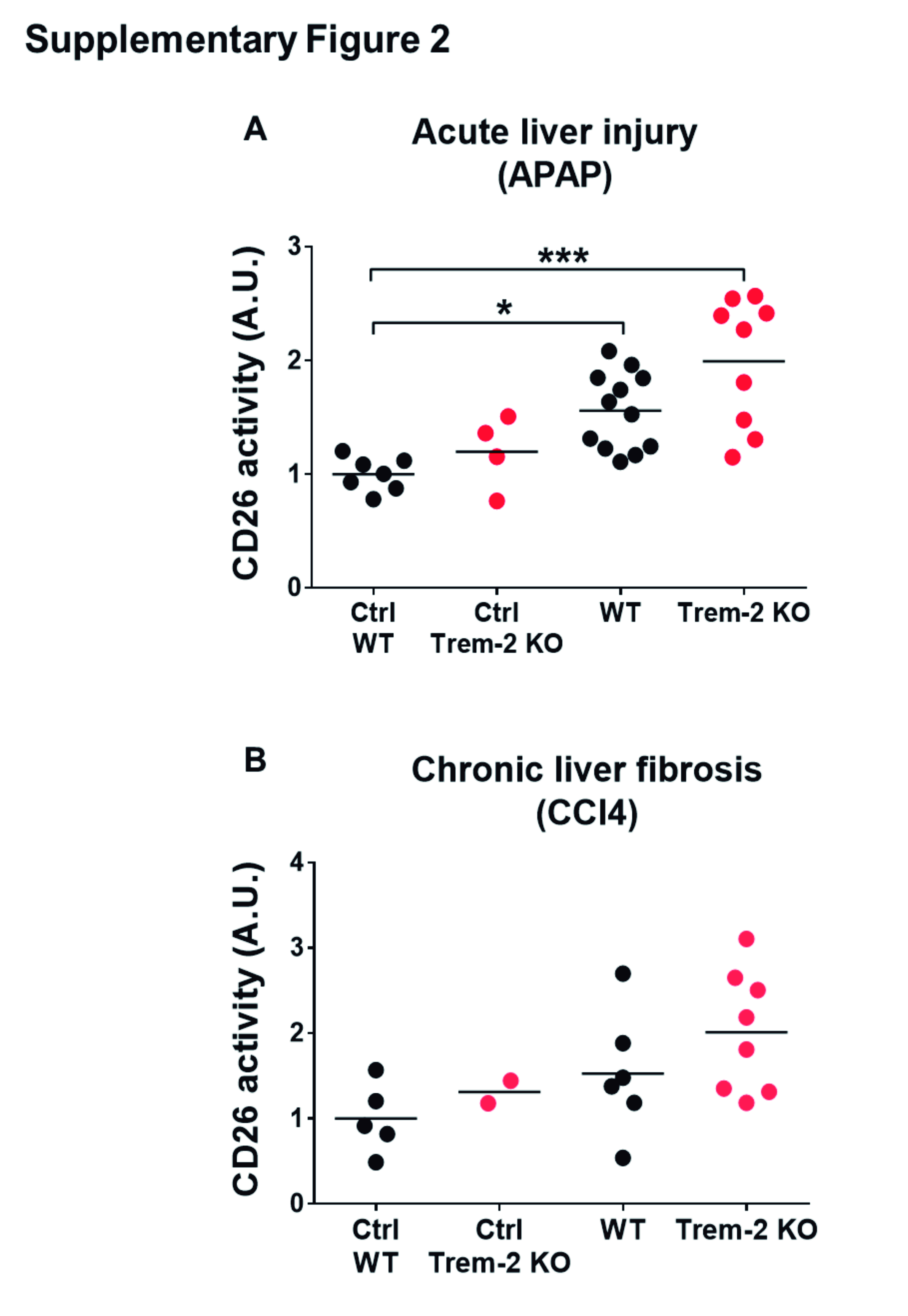

Supplement: Supplementary Figure 2 — Serum CD26 activity during recovery from acute liver damage and fibrosis regression. CD26 activity represented in arbitrary units (A.U.) was measured in the serum of wild-type and Trem-2 KO mice, during recovery from APAP (D3) (A) and during fibrosis regression (D3) induced by CCl4 (B) or in mice left untreated (Ctrl). Symbols represent individual mice. One-way ANOVA *, p<0.05 ***, p<0.001. [file Image_2.tif]

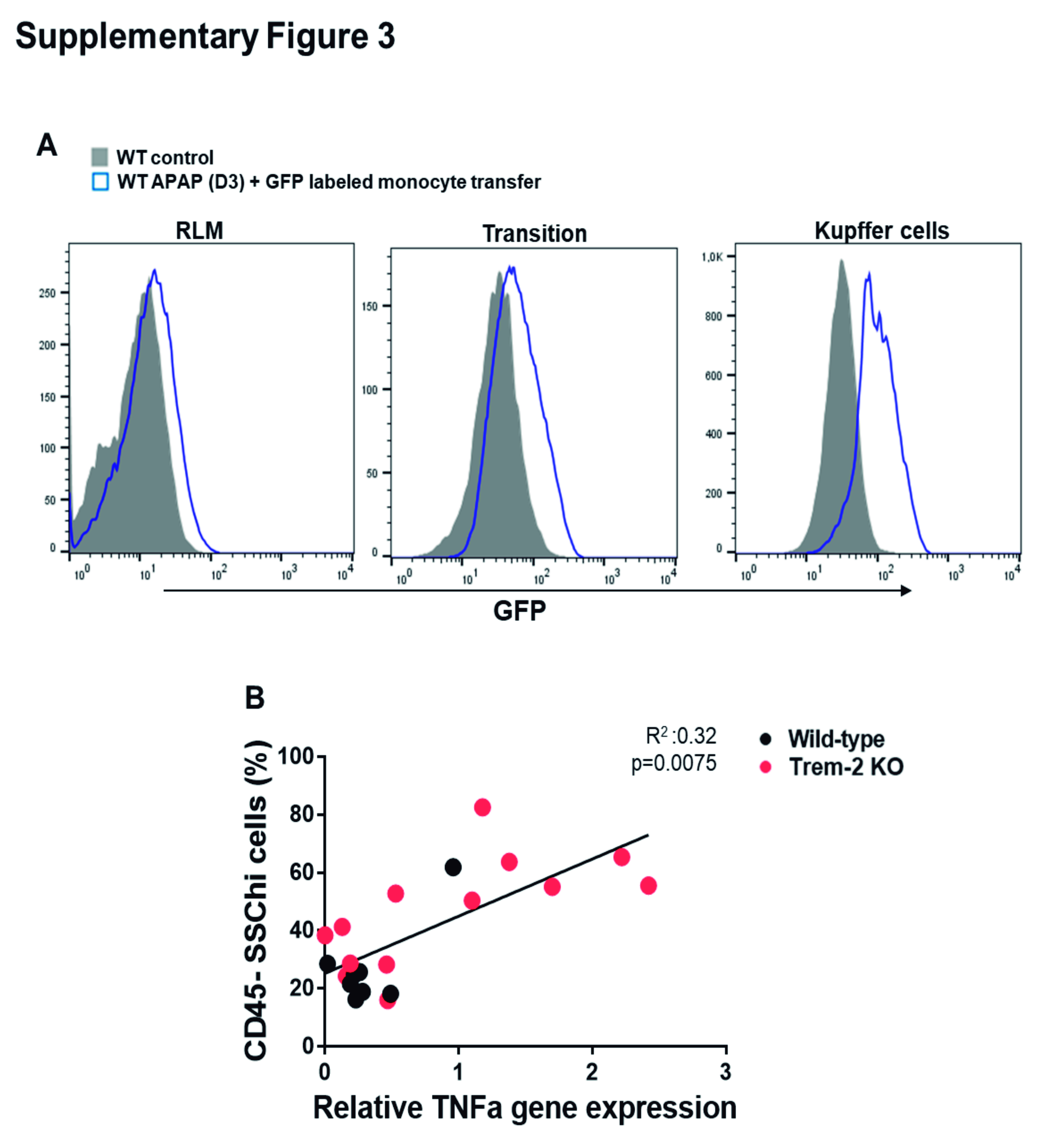

Supplement: Supplementary Figure 3 — Recruited monocyte tracking in liver macrophage populations following acute liver damage. Monocytes from B6.Actin-GFP mice were intravenously injected 12 hours after APAP treatment in wild-type mice and non-parenchymal cells were isolated at APAP-D3. Flow cytometry histograms show enrichment in GFP+ cells compared to non-transferred mice in different macrophage populations: RLM, Transition and Kupffer cells. [file Image_3.tif]

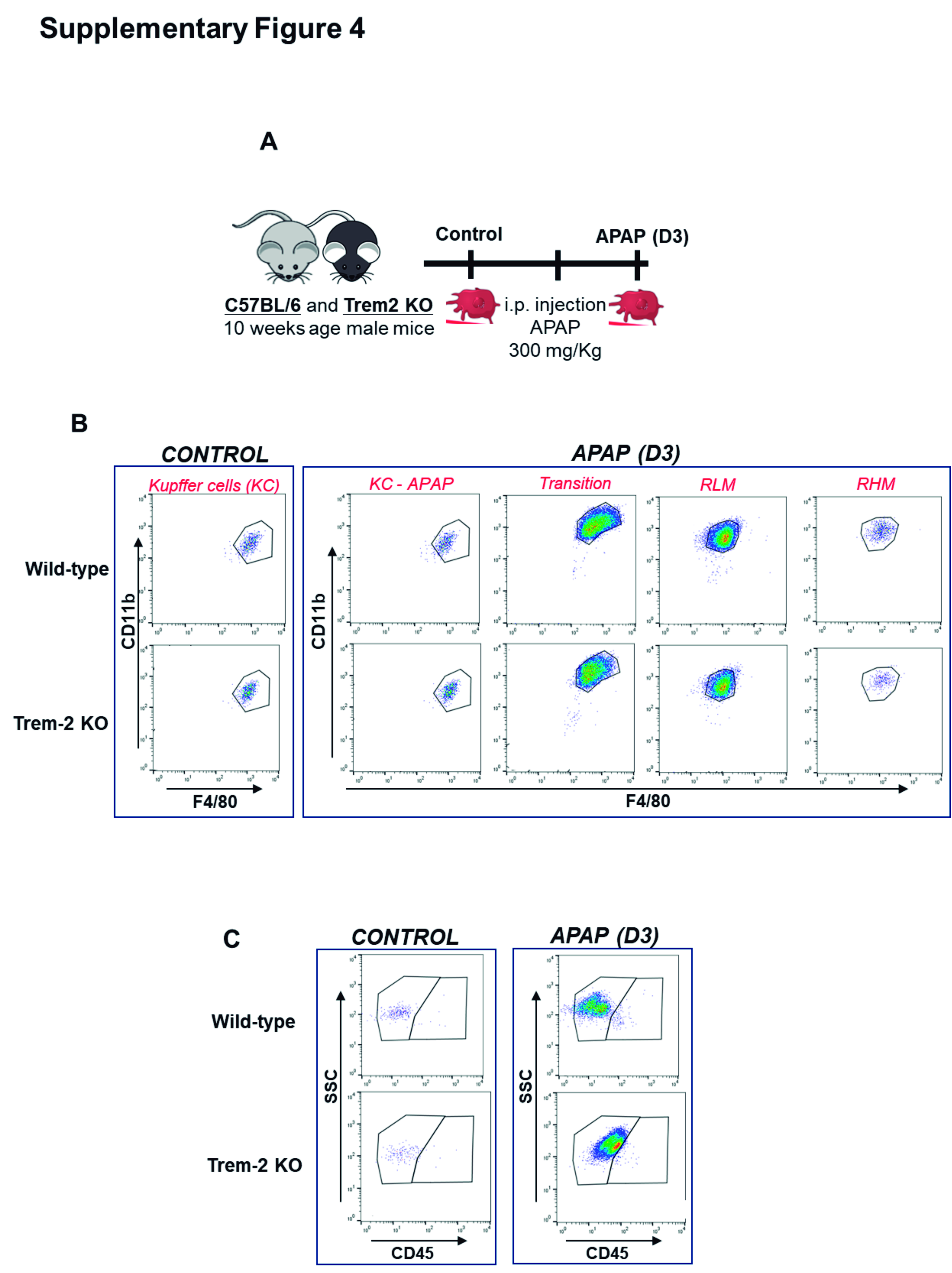

Supplement: Supplementary Figure 4 — Accumulation of CD45neg SSChi cells correlates with expression of pro-inflammatory cytokines in the liver. Positive correlation between frequency of CD45neg SSChi population and TNFα gene expression in liver non-parenchymal cells during recovery from chronic injury (CCl4-D3). Statistics: Pearson’s correlation test. [file Image_4.tif]

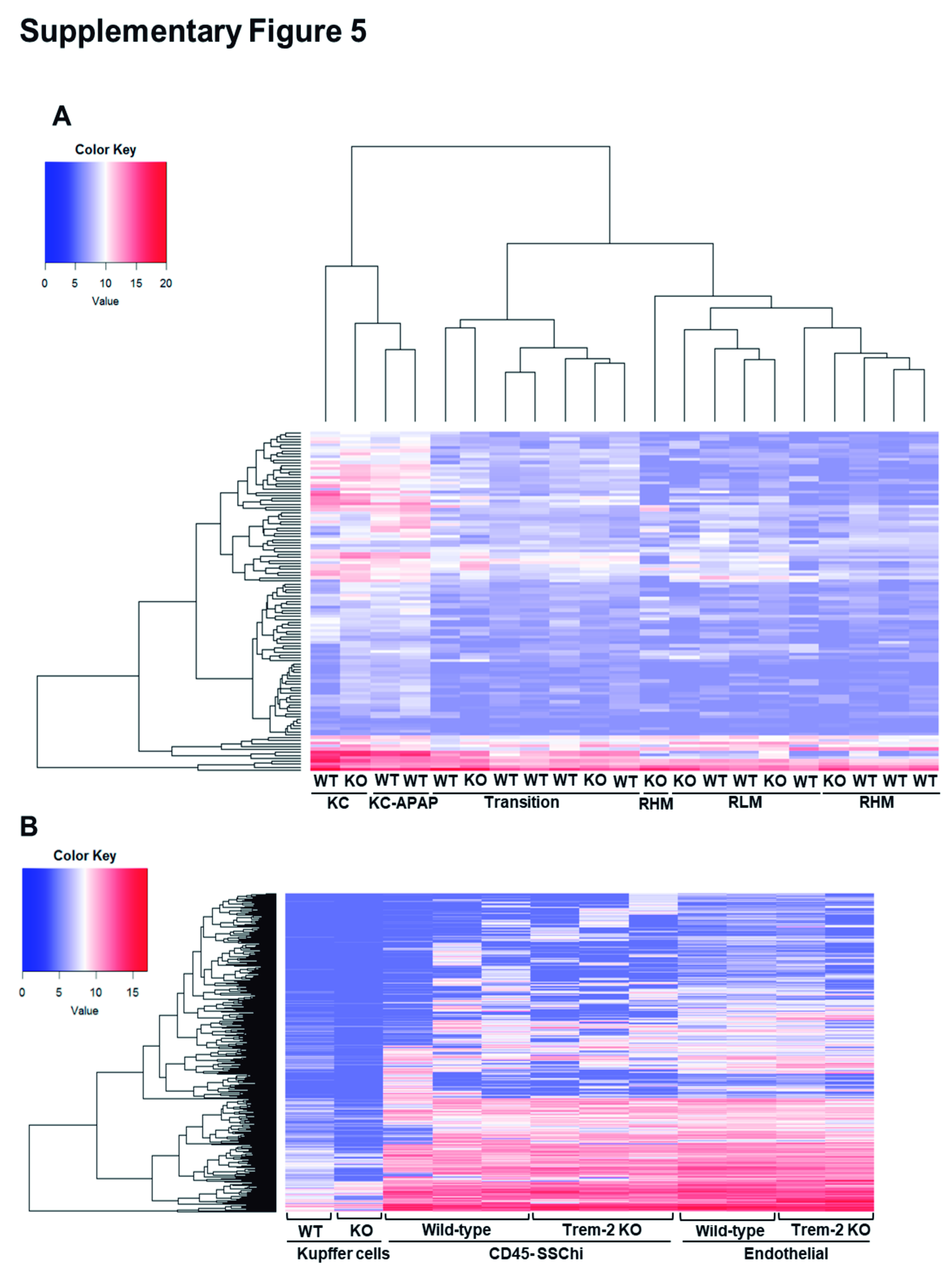

Supplement: Supplementary Figure 5 — FACsort-purified macrophage populations and CD45neg SSChi cells for transcriptomic analysis. Wild-type and Trem-2 KO mice received a single intraperitoneal injection of APAP and macrophage populations and CD45neg SSChi cells were sorted at APAP-D3 and in control mice. Macrophage populations were identified using CD45, Ly6c, CD11b and F4/80 markers and the purity of each population after sorting is shown (A). CD45neg SSChi population was identified using CD45 marker in APAP-D3 and in control mice and the purity after sorting is shown (B). Each sample for transcriptomic analysis was obtained from pools of 4 mice. [file Image_5.tif]

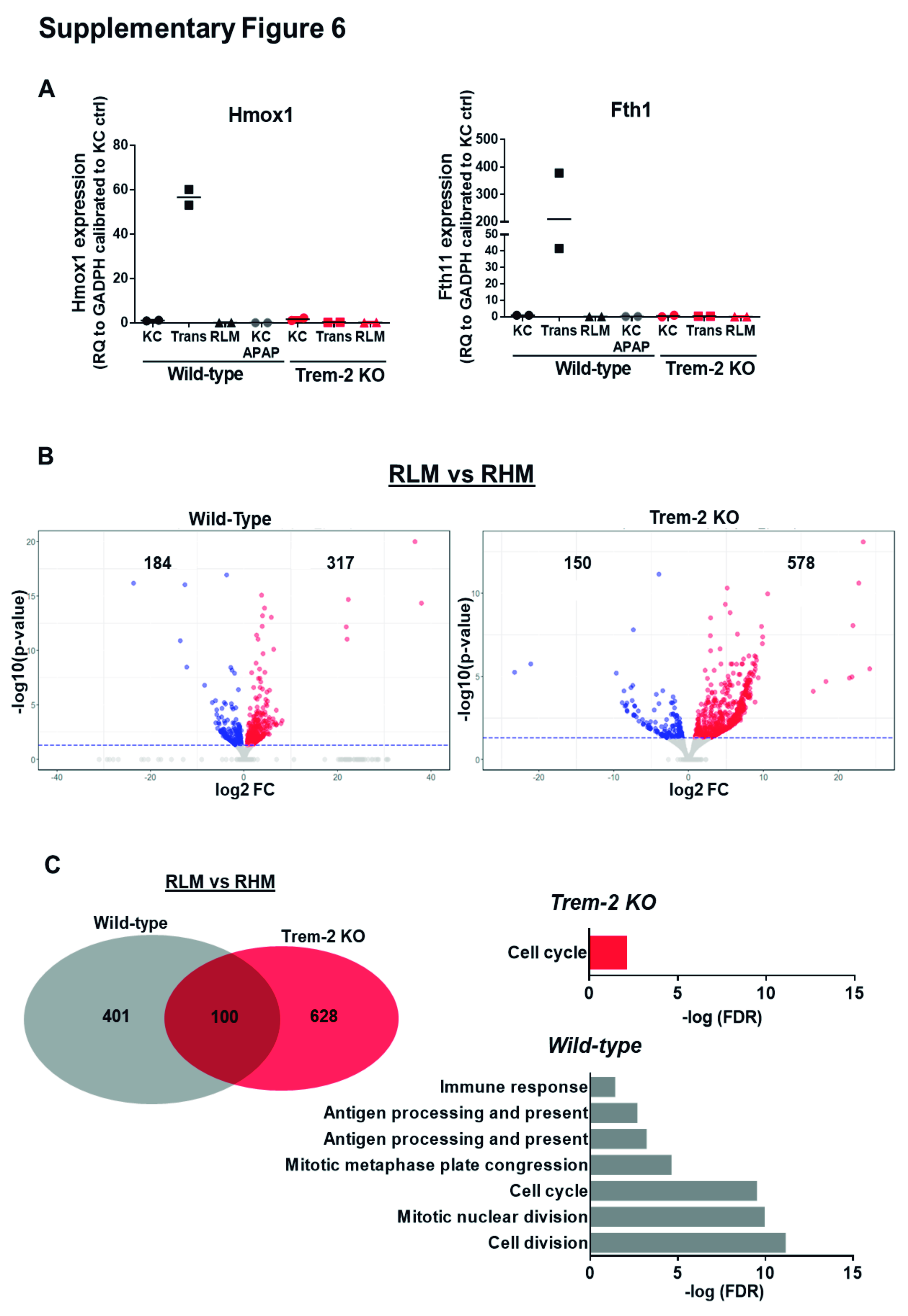

Supplement: Supplementary Figure 6 — Heatmaps of DE-genes representing hierarchical clustering of macrophage and CD45neg SSChi cell populations. Heatmap represents 100 KCs associated genes42 for different macrophage populations (A). Heatmap showing differentially expressed (DE) genes in CD45neg SSChi cells using KCs as reference: 821 genes upregulated in APAP-D3, 117 upregulated in control and 428 common to APAP-D3 and control as identified in Figure 2 .6A (B). [file Image_6.tif]

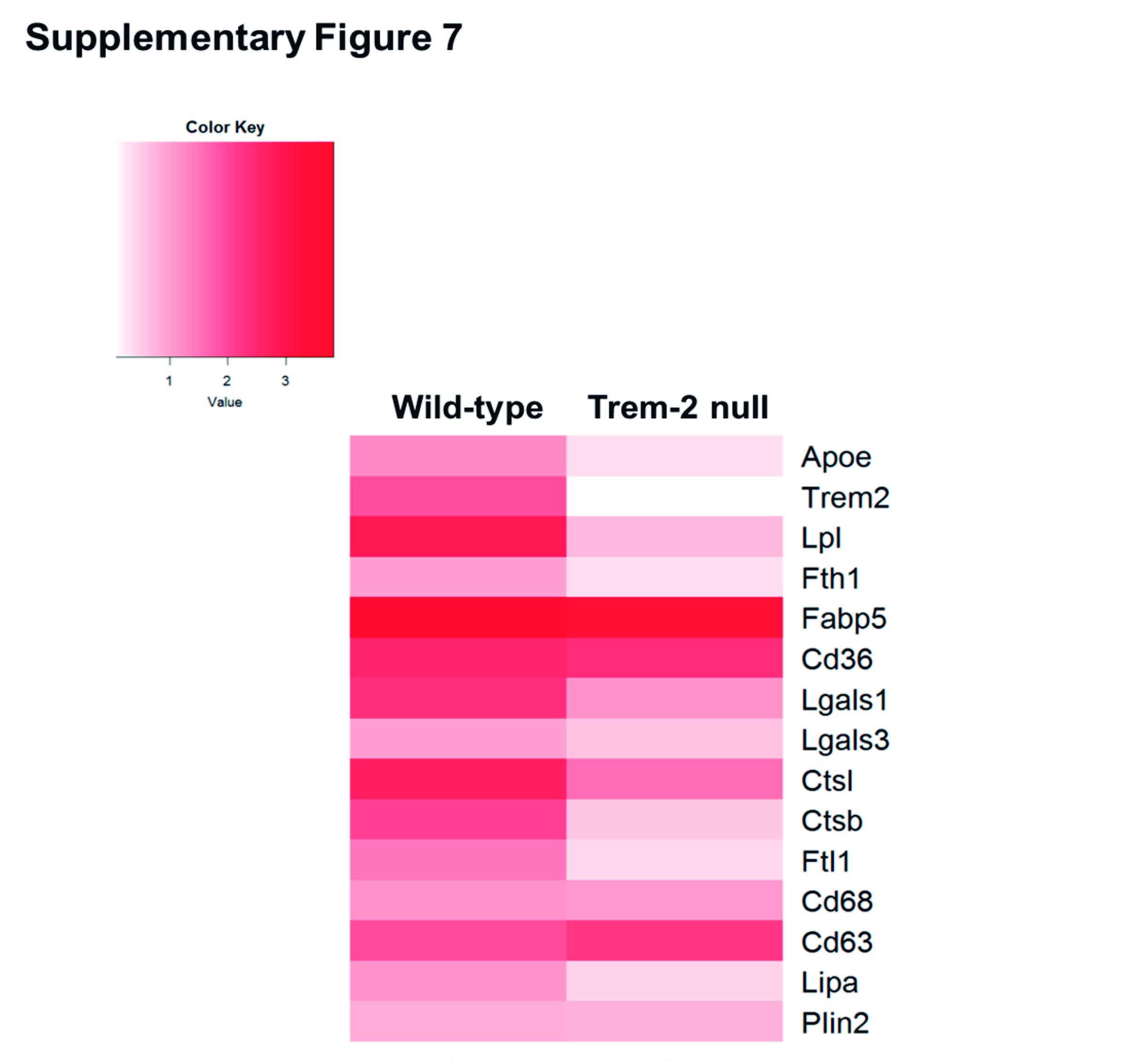

Supplement: Supplementary Figure 7 — Gene expression and transcriptomic analysis of macrophage populations. Hmox1 and Fth1 gene expression was evaluated by qPCR in sort-purified KCs, transition and RLM populations from wild-type and Trem-2 KO mice at APAP-D3 (A). Volcano plots representing differential expressed (DE) genes (q<0.05) between RLM and RHM in wild-type and Trem-2 KO mice. Red dots represent upregulated genes with LogFC>0, while blue dots represent downregulated genes with LogFC<0 significant for q<0.05 (B). Venn diagram representing DE-genes between RLM and RHM which are common to wild-type and Trem-2 KO (middle), exclusive for wild-type (left) or Trem-2 KO (right). Gene Ontology (GO) enrichment analysis in the ‘Biological Process’ category for DE-genes in wild-type mice and Trem-2 KO mice (C). Heatmap illustrating genes regulated by Trem-2 in transition macrophages. Log fold change (logFC) of genes previously associated to Trem-2 transcriptional signature (21, 22). Heatmap represents DE-genes upregulated in transition versus RLM at APAP-D3 in wild-type and Trem-2 KO mice [file Image_7.tif]
